# Inequality in abundance

**DOI:** 10.3389/frma.2022.980677

**Published:** 2022-07-29

**Authors:** Stephanie Plamondon

**Affiliations:** J. Reuben Clark Law School, Brigham Young University, Provo, UT, United States

**Keywords:** psychology, inequality, scarcity, social harm, technology

## Abstract

With technological advance has come the possibility of a new era of abundance. Technologies like 3D printing and robotics promise to lower the costs of production and distribution of goods and services, presumably making these goods and services readily available to those across income and wealth spectrums. This undoubtedly is a good thing. But what will be the effect of these technologies on existing wealth inequalities and the psychological and societal burdens they impose? Can we expect that this newfound abundance will help remedy the current historic levels of inequality in the U.S. and other western countries? Unfortunately, the answer is likely no, for two reasons. First, history suggests that inequality often persists even under conditions of abundance due to dynamics of power and politics and ongoing impacts of structural inequalities. Having more than enough of a particular good or service to go around does not guarantee that all will have access to this good or service. Second, even if the new abundance enabled by technology extends into all levels of the socioeconomic spectrum, enabling individuals to access goods and services (and their attendant benefits) previously beyond their reach, the harms that attend unequal societies will persist. Increasing evidence suggests that these harms, including increased violence and decreased health, arise not from access (or a lack thereof) to particular goods and services, but from the adverse psychological consequences of living in an unequal society. This is a psychological burden shared not just by those at the losing end of the inequality equation, but also those who enjoy a relative advantage in society. Unequal societies are psychologically harmful to all who live in them, regardless of where these individuals fall on the socioeconomic spectrum, and largely independent of the particular goods and services they enjoy. The upshot is that society cannot rely on new abundance technologies to automatically solve problems of inequality and the social and psychological burdens that plague those who live in unequal societies. Indeed, depending on how society responds to questions of access to these technologies, their introduction might exacerbate various forms of inequality. In light of this, it is crucial to address conditions of inequality head-on, so that the new era of abundance promised by technological advance can lead to real gains in individual and societal wellbeing.

## Introduction

We live in a world of scarcity. Resources, including goods and services, are limited. The restricted nature of goods and services, in turn, leads to higher prices and lowered access (Bakkeli, 2020). This is a reality human societies have lived with for many centuries.

Another historical feature of human societies is inequality (Jonsson et al., 2019). Scarce resources have always been distributed unequally to some extent—at various times and in various societies more or less unequally than others. By some accounts, the United States today is experiencing some of the highest levels of inequality seen since at least the beginning of the twentieth century, and perhaps beyond. Indeed, according to one economist, the level of inequality in the United States today is “probably higher than in any other society at any time in the past, anywhere in the world” (Piketty, 2014).

These two human realities—scarcity and inequality—in many ways go hand in hand. Scarcity may be one source of inequality–if there is not enough of a resource to go around, some will inevitably end up benefitting more than others. Yet technological advance provides the potential for reducing or even eliminating at least some forms of scarcity. For example, the Internet has ushered in a new era of abundance for informational and creative content by reducing the price of reproduction and distribution of this content to near-zero (Lemley, 2015). Other technologies offer the same promise for physical goods and services. 3D printing, for instance, will almost certainly make abundant a wide variety of physical goods as the technology advances and the price of producing and distributing these goods drops. And robotics technologies may do the same for services as robots become increasingly able to perform, cheaply and effectively, the bulk of services currently performed by humans (Lemley, 2015).

If this projection of reduced scarcity in the realm of goods and services is correct, what will this mean for inequality? When scarcity is mitigated, will inequality be tempered as well? It is tempting to think so. After all, if the newfound abundance of goods and services means that these become readily available to most members of society at low cost, at least one potential source of inequality (the differential ability to access particular costly goods and services based on income and wealth) goes away. And with that departure, ideally, the harms that attend unequal societies—harms ranging from slowed economic growth to adverse health and psychological effects on the society's citizens (Buttrick and Oishi, 2017)–would be mitigated as well.

But this hope is, unfortunately, unlikely to be realized—at least without significant planning and intervention. In the second Part of this chapter, I explain why. First, history belies the assumption that inequality necessarily disappears under conditions of abundance. Different societies at different times have enjoyed relative periods of abundance, and yet inequality has persisted (Jonsson et al., 2019). Scholars have understood this historical truth as confirming the hypothesis that inequality is as much about politics and power as it is about physical limits (Jonsson et al., 2019).

Second, with respect to the many harms that attend unequal societies, these harms may not have as much to do with access to goods and services as they do with psychological factors. For example, an increasingly accepted hypothesis put forward to explain the correlation between inequality and the raft of social harms that attends it posits that these harms arise from the adverse psychological consequences of living in an unequal society (Buttrick and Oishi, 2017). These consequences affect not only those at the bottom of the wealth and income ladder, but also those at the top. They arise from inequality itself rather than any objective measure of poverty or standard of living (Payne, 2017).

Third, somewhat paradoxically, certain conditions of abundance may bring with them their own psychological and societal harms—especially if it is not clear to all that the abundance is being fairly distributed. Reminders of abundance, for instance, might increase psychological distress related to concerns about fairness in ways similar to actual inequality (Gino and Pierce, 2009).

The implications of these insights about abundance and inequality are many, and in the third Part of this chapter I focus on two. First, given the political nature of inequality, we cannot automatically assume that a new abundance of inexpensive goods and services enabled by technology will necessarily translate into widespread access to this abundance. Structural inequalities can lead to bottlenecks that prevent the disadvantaged from accessing even those goods and services that in theory should be within their reach (Jonsson et al., 2019).

Second, even if all members of society can access newly abundant goods and services equally, to the extent that other forms of inequality (like structural, wealth, or income inequality) remain, many problems that plague unequal societies like the U.S. will persist. These problems grow from the psychological effects of inequality rather than any ability to access particular goods and services, even if these goods and services have real welfare-enhancing effects.

Given these implications, I ultimately conclude that scholars and policymakers must consider how to address the structural and political barriers that might prevent widespread access to the influx of inexpensive goods and services that will improve people's lives. But, more than this, if society wishes to tackle inequality and the social problems that come with it, it must consciously dismantle inequality in all its forms.

## Inequality: Definitions and harms

Scholars have predicted a technological revolution that will change our experience of scarcity. Advances in technologies like 3D printing, robotics, and synthetic biology will lead to a new infusion in the market of low-cost goods and services—much like the Internet has done for information and creative content—making these products, in economic terms, abundant rather than scarce (Lemley, 2015).

Living in a society rife with inequality—as those of us in the United States do—the question arises as to whether the specific promise of abundance offered by these emerging technologies could positively impact this situation.

But before asking if this will happen, we might first ask if (and why) we want it to. Relatedly, we should clarify what we mean when we talk about inequality.

### Defining inequality

Neither of these are easy questions to answer, and I do not attempt to do so comprehensively in this chapter. As for the latter question—what we mean when we refer to inequality—scholars have taken a range of approaches (economic, social, philosophical, and others) to address it. For purposes of this chapter I use the term to refer broadly and generally to an unjust distribution of opportunities and resources within a society (Koh, 2020). Inequality is not necessarily present merely because resources and opportunities are distributed unequally—the justness, or fairness, of the distribution is a relevant consideration under the definition I adopt (Buttrick and Oishi, 2017). Justness is, however, often difficult to gauge, as will be discussed further below (Gino and Pierce, 2009). And perceptions of inequality can be equally significant for their ability to give rise to particular social harms as the objective presence of the phenomenon. Further, although absolute levels of distribution are not necessarily determinative of the presence or absence of inequality, the wider and more visible the gap between the haves and have-nots in a particular society, the less likely it will be that distributions are just in fact, and the more likely the gap will be perceived as unjust. Thus, under my definition, inequality manifests when there is an actual or perceived unjust distribution of resources and opportunities, and this is more likely to obtain when the distribution is clearly uneven among citizens or groups.

### Inequality's harms

The other question—whether we want to eliminate inequality, and if so, why—is a normative one; as with all normative questions, opinions as to the correct answer can differ. Here, I advance the view that inequality (especially extreme inequality of the kind we are currently experiencing in the United States) is normatively undesirable, and I offer some reasons to support this position.

First, there is the simple fact that the unfairness of inequality *feels* wrong to many people. Humans have a finely tuned sense of fairness that arises very early in childhood (Yang et al., 2014). Subjective perceptions of fairness are rooted in conceptions of human dignity, a value many find compelling and desirable (Organ and Moorman, 1993). When the values of dignity and equality are not respected in a society, it causes subjective discomfort among its citizens (Buttrick and Oishi, 2017). More concretely, inequality gives rise to measurable psychological harms. Residents of unequal societies are less happy, exhibit more mistrust and increased anxiety, and have higher rates of depression than those in more egalitarian societies (Messias et al., 2011; Ferrer-i-Carbonell and Ramos, 2012; Buttrick and Oishi, 2017).

Perhaps even more concerning than the psychological harms associated with inequality are the social harms that manifest themselves in unequal societies. These harms are wide-ranging, and include, among other things, increased violence, decreased health, reduced life expectancy, higher infant mortality, lower social cohesion, weaker governance, poorer educational attainment, slower economic growth, and lower social mobility as compared to more equal societies (Buttrick and Oishi, 2017; Coccia, 2018; Wilkinson and Pickett, 2018).

In light of these psychological and social harms, the normative case for eliminating inequality is a strong one. In the next Part, I explore how the expectation of newly abundant goods and services, made possible by technological advances, might contribute to this venture.

## Inequality and abundance

In a society of abundance of particular goods and services, one might hope and expect—perhaps for some of the reasons articulated above—that inequality will be mitigated. But will it? In this Part, I address this question. I explain why we should not expect this salutary result without significant policy intervention—and in fact, why without such intervention, the newfound abundance might exacerbate current problems of inequality. I focus first on the political aspects of inequality before addressing the psychological impacts of living in unequal societies—impacts which give rise to a wide range of additional social harms. Each of these frames—the political and the psychological—helps elucidate the limits of abundance of goods and services as a catalyst for eliminating inequality.

### The political nature of inequality

One might think of inequality as a problem that arises, at least in part, from scarcity. Definitionally, a scarce resource is one that is incapable of meeting demand (Merriam-Webster, 2020). The seemingly inevitable consequence of this is that some will be able to obtain the resource while others will not, leading to inequality.

Extending this line of thinking, one might conclude that once a particular resource is no longer scarce, that resource will cease to be a source of inequality. When there is more than enough of a resource to go around, principles of economics dictate that the cost of this resource will approach zero, making it theoretically available to any who desire it (Lemley, 2015).

Contrary to this assumption, however, scholars have highlighted the phenomenon of “scarcity amid abundance” (Jonsson et al., 2019). It manifests when some segments of a society experience functional scarcity even when a resource is abundant (in the sense that there is enough to go around). Economists, sociologists, and historians have documented this phenomenon in various societies at various historical time points. For example, Amartya Sen has described how famine events have taken place in various societies even when there was enough food to adequately provide for everyone (Sen, 1981). And Elizabeth Chattergee has explained that India now finds itself in an unfortunate situation with respect to energy, where some groups in the country enjoy a surplus of the resource while others go without (Chattergee, 2019).

The phenomenon of scarcity amid abundance suggests that even abundant resources might be significant sources of inequality. But why?

One potential answer lies in the political nature and power dynamics of inequality. Political and power considerations complicate the market forces that would otherwise lead to widespread access to an abundant resource. In some cases there might be a concerted effort among the powerful in a particular society to withhold resources from those with less power. This effort may arise even in times of abundance because the powerful have a distorted sense of their personal need; or they might fear that the abundance will not endure. For example, David Lamoureux describes how the British in colonial Lagos hoarded land and water for themselves (Lamoureux, 2019). Or it could be a pure exercise of greed or power (Crawford, 2018; Jonsson et al., 2019). In other cases, the unequal distribution of abundant resources could be a result of simple indifference on the part of those in power; for instance, the lack of will on the part of the British to make the infrastructure investments necessary to provide colonial India with electricity (Chattergee, 2019).

Another possible explanation for scarcity amid abundance lies in the ongoing impacts of past inequalities. Past wrongs can give rise to ongoing structural inequalities that make it difficult to distribute abundant resources, even when there is a political will to do so. For example, Chattergee explains how the current energy inequalities in India can be traced in part to past colonial rule and Britain's lack of interest in providing India with electricity. Because energy is a resource that requires significant infrastructure investments, the impact of past neglect continues to reverberate today (Chattergee, 2019).

Scarcity amid abundance is not a phenomenon confined to other places and other times. Examples of inequality in the midst of abundance can be seen in the United States today. The problem of food deserts, for example—where some U.S. communities struggle with nutritional inequality—illustrates the phenomenon and underscores the point that even the most affluent societies can suffer from it (Walker et al., 2010; Allcott et al., 2018; Palazzolo and Pattabhiramaiah, 2020). Indeed, even Lemley's example of the Internet making information and creative content abundant (Lemley, 2015) demonstrates how the simple market equation of abundance leading to near-zero cost and widespread access can fail to accurately describe the reality on the ground. The fact is that many in the U.S. today are unable to partake in the abundance the Internet offers, perhaps in part due to the political power wielded by major private companies and a lack of will on the part of the government (Crawford, 2018).

The political realities of inequality suggest that a resource may not cease to be a significant source of inequality merely because it is theoretically (in economic terms) abundant. In Part III I explore what this means going forward as policymakers contemplate how to approach the coming wave of low-cost goods and services. But first, I examine how an understanding of the psychological underpinnings of inequality should also dampen any sanguinity about an abundance of goods and services automatically mitigating inequality. Instead, the challenge for the U.S. is to plan and prepare so that this coming abundance of goods and services can translate into increased welfare and serve as a vehicle for reducing inequality.

### The psychological and social impacts of inequality

As detailed above, numerous studies have identified the many social and psychological ills unequal societies face. But to understand whether a new abundance of goods and services will cure inequality and the harms that attend it, it is instructive to understand why these troubles arise in the first place.

Scholars are beginning to provide some answers to this question. The emerging picture suggests that the psychological and social difficulties associated with inequality share a causal relationship. Specifically, it appears that the social harms characteristic of unequal societies grow at least in part from the negative psychological influence inequality has on a society's citizens (Buttrick and Oishi, 2017).

#### Inequality and mistrust

How does this work? As explained, citizens of unequal societies suffer from a number of psychological impacts. One of these impacts is an increased mistrust of both other citizens and the system as a whole (Algan and Cahuc, 2013). People who live in societies where inequality reigns tend to regard their system of governance as unfair. They are also prone to suspecting that those at the top achieved their favored positions through unethical and dishonest means (Grosfeld and Senik, 2010).

This lack of trust leads to more tangible and destructive harms. For example, in an empirical study, Nishi et al. demonstrated how mistrust could impact community formation and ultimately, economic growth. In the experiment, which was set up as a modified public goods game, participants initially allocated a higher share of the wealth cooperated less and acted to preserve their wealth. Those with lower allocations were then forced to choose between being exploited by the “rich” or refuse to cooperate themselves. In games where initial allocations were unequal, mistrust flourished, cooperation faltered, and overall wealth generation was stymied, leading to slowed growth of the game's economy (Nishi et al., 2015).

Other research links the mistrust inequality breeds with the reduced civic participation and ties seen in unequal societies. Those who live in more unequal communities are less likely to participate in social clubs and service organizations, even when they have the ability and resources to do so (Alesina and La Ferrara, 2000; Lancee and van der Werfhorst, 2011). Inequality has been shown to be a major driver of this association, with the mistrust inequality engenders explaining the causal relationship (Costa and Kahn, 2003; Uslaner and Brown, 2005). This lack of civic engagement and social support might also help explain the causal relationship between inequality and mistrust on the one hand, and poor health outcomes, including increased mortality and reduced life expectancy, on the other. Frank Elgar, who has studied this phenomenon, hypothesizes that “[s]ocieties with low levels of trust may lack the capacity to create the kind of social supports and connections that promote health and successful aging” (Elgar, 2010).

Finally, there is ample evidence that mistrust prompts people to act in unethical and anti-social ways. When people feel that others are getting ahead unfairly, it is easier to rationalize their own unethical behaviors that attempt to even the playing field (Grosch and Rau, 2020). Cheating is therefore more prevalent in unequal societies (Neville, 2012). Inequality also gives rise to higher homicide rates, a causal relationship that is mediated by lowered trust in others (Elgar and Aitken, 2011).

#### Inequality and status competition

The ripples moving outward from inequality's stone throw of mistrust are serious and far-reaching. But inequality has additional psychological impacts on the people experiencing it. In addition to mistrust, inequality fosters feelings of envy and jealousy, feelings which, in turn, fuel status competition. Residents of unequal societies place more importance on people's relative positions on the social ladder compared to residents of more equal societies (Kraus et al., 2013; Paskov et al., 2013). They also act in ways that demonstrate the weight they place on status, working longer hours to get ahead and conspicuously consuming goods that signal their status to others (Bell and Freeman, 2001; Bowles and Park, 2005; Walasek and Brown, 2015).

Status competition may not at first glance appear to be particularly harmful in and of itself. After all, a motivation to work longer hours might very well improve productivity, and conspicuous consumption might be seen as a relatively harmless activity that also fuels economic growth. However, it appears that status competition generates extreme anxiety about one's relative position in society that leads to all sorts of negative outcomes, including worse health (caused by the negative effects stress has on the body), increased risky behaviors (as people become more willing to take ill-conceived gambles in order to increase their status), and higher levels of obesity and drug abuse (as people turn to these comforts to mitigate the stress and anxiety generated by status competition) (Wilkinson and Pickett, 2009; Mirsha et al., 2015; Payne, 2017).

One might reasonably assume that these impacts (stress, health problems, increased risk taking, and unhealthy behaviors) would affect only the “losers” in an unequal society. And to some extent, this is true. Those at the bottom of the status ladder in an unequal society experience at least some of these harms more intensely than those on higher rungs (Buttrick and Oishi, 2017). Remarkably, however, those at the top of the ladder are not immune from the stress of status competition and all the harms, including reduced life satisfaction, that flow from it (Layte and Whelan, 2014; Cheung and Lucas, 2016; Payne, 2017).

The hierarchy-spanning effects of status competition arising from inequality have been demonstrated nicely in the societal microcosm of an airplane. Airplanes, with different levels of seating classes and boarding groups, tend to replicate existing social hierarchies in very visible ways (Payne, 2017). Payne describes research by Katherine DeCelles and Michael Norton showing how these visible reminders of differential status can affect behavior. After analyzing data from millions of flights, DeCelles and Norton found that so-called “air-rage” incidents, in which a passenger behaved badly or caused a disturbance, were almost four times more likely to occur on flights that had a first-class section compared to flights that did not. The disturbances were almost twice as likely on flights where the economy-class passengers were forced to walk past the seated first-class passengers as they boarded as compared to flights where the economy-class passengers boarded in the middle or back of the plane (DeCelles and Norton, 2016).

As Payne points out, given the cost of an airline ticket, it is unlikely that a typical commercial flight has many truly poor passengers (Payne, 2017). Yet the status competition triggered by a plane's social hierarchy infected even the relatively well-off airline passengers. Further, the recorded disturbances were not limited to those at the bottom of the airplane's hierarchy. Although economy-class passengers were more likely to cause a disturbance, a significant percentage of incidents were caused by first-class passengers (DeCelles and Norton, 2016).

#### How the psychology of inequality informs expectations for abundance and inequality

Understanding the psychological and social impacts of inequality and how they are linked can help scholars evaluate the potential effects of a new influx of low-cost goods and services into an unequal society like the U.S.

First, to the extent that these goods and services do make it into the hands of the “have-nots” in our society, they undoubtedly have the potential to improve quality of life. A society that has better access across the wealth and income distribution to a variety of welfare-enhancing goods and services—including medical services—is almost certainly better off than a society in which only a subset of the population at the top of the social hierarchy has access to these things.

But whether this new abundance of goods and services will mitigate inequality and the harms that attend it is a different question altogether. The increased access to particular goods and services will certainly mitigate or eliminate one basis on which individuals might distinguish themselves in an unequal society—namely, the differential ability to enjoy these goods and services. But to the extent that other, more fundamental inequalities remain—inequalities in income, income mobility, wealth, access to education and a good job, ability to vote, incarceration rates, and others—a new abundance of goods and services may not do much to remedy the mistrust and status competition that, in turn, give rise to the myriad social problems observed in unequal societies.

This is true in part because, as research shows, it is not some objectively low standard of living that causes the mistrust and status competition associated with inequality. Indeed, even the relatively well-off airline passengers in the airplane study were not immune from inequality's psychological sway. As Payne notes in an article discussing his research, even the poor in the U.S. have access to a variety of goods that might have been unattainable for them 20 years ago—including TVs, cell phones, and microwaves (Kolbert, 2018). And yet, the problems typical of unequal societies are keenly felt in the U.S. By way of explanation, Payne offers that “[i]nequality makes people *feel* poor and *act* poor, even when they're not. Inequality so mimics poverty in our minds that the United States, the richest and most unequal of countries, has a lot of features that better resemble a developing nation than a superpower” (Payne, 2017).

As an aside, this feeling of being poor, despite all evidence to the contrary, may perhaps help explain why so many extremely wealthy people in the U.S. (more than 96% of millionaires who belong to the wealthiest 10% of citizens in the country) classify themselves as “middle class.” These individuals might genuinely feel that they are not particularly wealthy, in part because they are comparing themselves to those who have even more than they do (Frank, 2015; Payne, 2017; Kolbert, 2018). For example, in her research into inequality, sociologist Rachel Sherman interviewed a woman with a household income of over two million dollars a year who described herself as middle class. In explaining her reasoning for this categorization, the woman stated that “no matter what you have, somebody has about a hundred times that” (Kolbert, 2018).

Near-universal access to televisions and cell phones, while arguably making life easier and better for citizens, has done little to solve problems of inequality in the U.S., including the psychological impacts of status competition and mistrust that give rise to even greater social harms. It would be naïve to assume that access to *more* goods and services alone, without an attempt to address underlying issues of inequality, would have any different effect.

This is particularly true given that in societies where income and wealth inequality reign, citizens invest more of their effort and money into signaling their status through the conspicuous consumption of positional goods (Walasek and Brown, 2015). Positional goods are intended to signal one's position or status in a society. They are scarce—usually made intentionally so by those offering them—and therefore presumably available only to high status individuals with great wealth. Examples of positional goods include brand name items, rare and expensive sports cars, and tickets to high profile sporting events like the Super Bowl. Even as new technologies make a variety of new goods and services available to citizens across the wealth and income spectrums, as long as wealth and income inequality remain there will be status competition that plays out in part through the acquisition of positional goods—which despite technological advance will continue to remain scarce either naturally (as in the case of Super Bowl tickets, for which there will always be a limited number) or through the efforts of those offering them (for example, through the use of high cost brand names or limited product runs). In fact, as certain goods and services, because of their newfound abundance, lose the power to signal status, we might expect the development of new vehicles for signaling status. Non-fungible tokens, or NFTs, might be one example of this (Fairfield, 2022).

Policymakers should not expect, therefore, that simply increasing access to a variety of goods and services will address the larger problems that arise from inequality—though these goods and services might indeed make people's lives better in measurable ways. As long as income, wealth, and other forms of inequality remain, they should expect that mistrust and status competition will continue to flourish, leading to the raft of additional social harms seen in unequal societies like the U.S. In fact, there is intriguing initial research suggesting that a backdrop of abundance might exacerbate the negative feelings that characterize unequal societies, making the harms that arise from these feelings even more likely to occur.

### How perceptions of abundance might exacerbate the psychological impacts of inequality

In the previous Section Inequality: Definitions and harms explained why a newfound abundance of goods and services, made possible by new technologies, is unlikely to remedy the negative psychological effects of living in an unequal society. But it is plausible to think that this abundance might at least mitigate these psychological effects somewhat. After all, if most people are newly able to get more of what they need, they may become less concerned with what they do not have, which in turn might open the door for them to trust more and compete less.

Interestingly, however, a series of studies by Francesca Gino and Lamar Pierce suggest that this might not be the case, and that in fact, a setting of abundance might have the opposite effect—increasing mistrust, envy, and some of the other negative feelings common in unequal societies.

Building on work finding that the presence of wealth may encourage people to engage in unethical behaviors, Gino and Pierce set out to study how a context of abundance might affect people's behaviors in an experimental setting (Gino and Pierce, 2009). In the study they define abundance as “a large pool of visible resources that are either shared by [societal] members or possessed by individuals within the [society].” Subjects in the study were asked to complete a word task and were given a pile of cash from which to pay themselves based on their performance. In the “abundance” condition, participants were given the cash from a table containing much more money than was necessary to pay all participants, whereas in the “scarcity” condition participants were given funds from a table that contained only enough cash to pay the participants. The researchers found that the abundance condition produced twice as many cheaters—participants who overstated their performance in order to pay themselves more than they had earned—than the scarcity condition. The magnitude of the cheating—i.e., the level of overstatement—was also significantly higher in the abundance condition.

In subsequent studies, Gino and Pierce set out to determine what might be prompting the unethical behaviors seen in conditions of abundance. They examined a number of hypotheses, including the possibility that the cheating was mediated by feelings of envy based on a perception of inequity triggered by the abundant cash. And in fact, the authors did find that envy was a prime motivator of the cheating, while alternative hypotheses, like simple greed or participants' perceptions that their actions would harm others less in the abundance condition, were not supported (Gino and Pierce, 2009).

This series of studies by Gino and Pierce has not been the subject of subsequent research, so the results should not be overstated. However, their findings do dovetail nicely with the psychology literature on inequality discussed above and provide some insights into the feelings and behaviors prompted by conditions of abundance. As explained above, feelings of envy triggered by conditions of inequality can cause people to mistrust others and believe that these others are succeeding unfairly. These perceptions in turn, can lead to unethical behaviors as people rationalize their own attempts to get ahead (Grosch and Rau, 2020). The so-called “abundance effect” identified by Gino and Pierce suggests that similar feelings and behaviors might be prompted by the mere presence of abundance, which, absent any evidence to the contrary, can give rise to *perceptions* of inequity.

Extrapolating from the lab to the real world, what might this mean for a situation in which a new abundance of goods and services is introduced into a highly unequal society like the U.S.? As explained above, that event alone is unlikely to remedy the psychological harms that flow from living in conditions of inequality. But, more than this, the new abundance of goods and services—especially if it is not clear that everyone is benefitting equally from it—could exacerbate the existing negative feelings engendered by inequality or trigger additional adverse emotions, as the new visible reminders of abundance activate people's sense that they are not getting their fair share.

Further, as explained above, these emotions and behaviors associated with feelings of inequity are causally linked to a wide range of social harms, including increased violence, worsened health, and slower economic growth. Rather than expecting the new abundance of goods and services to remedy these problems, there is reason to believe that it might worsen them absent significant policy intervention.

## Implications

The above discussion of the political and psychological forces underlying inequality leads to two major conclusions about how a new abundance of goods and services can be expected to impact an unequal society like the U.S. First, the fact that these goods and services will become theoretically abundant does not necessarily mean that they will be abundant—i.e., widely available across income and wealth distributions—in practice. And second, even if the new abundance of goods and services proves in fact to be accessible to all, this will not automatically mitigate inequality and the social problems that grow from it. It might even exacerbate these problems by further triggering the psychological forces that give rise to them. In this Part, I explore what actions should be taken if policymakers want the coming abundance to offer real gains to the wellbeing of citizens on all rungs of the social ladder.

### Ensuring access to abundance

Economic theory predicts that when a resource is abundant (i.e., there is more than enough of it to fill demand) the cost of this resource will approach zero, making it theoretically available to all who desire it. As demonstrated by the phenomenon of “scarcity amid abundance”, however, this prediction often fails to be realized in practice. The reasons for this are myriad, but as discussed above, they can include hoarding by those in power, the absence of distributional infrastructure, the influence and greed of small but powerful interest groups, or a lack of will on the part of decision-makers.

What does this mean for policymakers who hope that a new influx of goods and services brought about by technological change can be enjoyed by all citizens? The first lesson is that this might not happen without identifying and eliminating potential barriers to access.

For example, Lemley notes that the Internet has made informational and creative content abundant in the economic sense (Lemley, 2015). Yet, it is not abundant in the practical sense because large swaths of the population—about 18% of African American households, among others—do not have home Internet and so are unable to easily access this content (Crawford, 2018) (though it is true that these numbers look much better if you consider cellular internet access). Susan Crawford identifies cost as the major driver of this lack of access, and points to a lack of competition and government oversight of Internet service providers as the underlying culprits (Crawford, 2018). According to Crawford, the way to make this content truly abundant in both the economic and practical senses would be for the government to invest in the necessary infrastructure and then allow private actors to use this infrastructure to compete for consumers (Crawford, 2018).

Scholars predict that 3D printing will lead to an abundance of goods in the same way that the Internet has led to an abundance of content (Desai and Magliocca, 2014; Lemley, 2015). According to Lemley, for example, the day may soon arrive when most citizens will have access to 3D printers in their homes or public facilities and will be able to manufacture a variety of desired goods with widely available online designs (Lemley, 2015). This prediction might in fact be more easily realized than the goal of universal home Internet access, since (unlike the Internet) 3D printers do not require costly infrastructure that can hinder competition. As 3D printing technology improves and more companies enter the market, then, it is quite possible that the cost of owning a 3D printer will drop to the point where most homes will have one, just as most homes in the U.S. now have a personal computer (Lemley, 2015). However, that scenario is not necessarily a given, and it could also be the case that the cost of 3D printers will remain high for a significant amount of time, leading to disparities in who can take advantage of their manufacturing abilities. This disparity could in turn exacerbate existing inequalities as those most in need of what 3D printing has to offer are the least able to access it. Policymakers might therefore consider what could be done in this latter scenario to ensure equal access to 3D printing across wealth and income distributions. For example, though Lemley talks about 3D printing being available in “public facilities,” this is a scenario that will require planning and funding to be realized. That said, it should be relatively straightforward for the government to provide funds to ensure that 3D printers are in fact available and accessible in libraries and other public places. Desai and Magliocca also discuss government interventions that can be undertaken to ensure that people—once they do have access to 3D printers—can take full advantage of what the technology has to offer, including creating intellectual property infringement exemptions for small-scale printing activities and establishing a notice-and-takedown-based safe harbor for websites hosting files with 3D printing instructions so that these files can also be widely accessed (Desai and Magliocca, 2014).

A similar analysis holds for Lemley's prediction that robots will be able to do for services what 3D printers will do for goods, completing tasks like serving meals, cleaning houses, and driving cars. The challenge for policymakers is in ensuring that all households have equal access to these technologies. Given the expectation of the kinds of tasks these robots will eventually perform, it will not be enough, as it might be with 3D printers, to have these robots available at public facilities. Individuals must have access to these technologies in their own homes. Government subsidies—for example in the form of tax rebates—could help ensure that these important technologies become widely available.

In contrast, Lemley's predictions about synthetic biology might look more like the Internet scenario due to the presence of mediators and gatekeepers. For example, Lemley hypothesizes that advances in genetic engineering, when combined with 3D printing, will allow for medical offices to “generate custom genes to order” and create organisms and body parts in-house (Lemley, 2015). However, as the current state of medical care in the U.S. teaches us, the fact that a doctor's office or hospital can do something cheaply and easily does not necessarily translate into better access to these services across the population. The U.S. lags behind other countries in access to affordable health care, which is hypothesized to result in part from a lack of universal insurance coverage (Osborn et al., 2016). Unsurprisingly, this lack of access hits those at the lower end of the income spectrum hardest (Millman, 1993). The fact that medical providers may be able to offer advanced services at a lower cost to them will not, therefore, guarantee that all members of the population will be able to affordably access these services. In fact, this possible future state of affairs may end up exacerbating existing inequalities, as those who already have access to medical services will be able to take advantage of even more advanced technologies, while those without access will be left in the cold. And though Lemley entertains the possibility of a time where individuals may be “printing [their] own organisms”, most of us will likely be depending on medical intermediaries for these kinds of services for the foreseeable future (Lemley, 2015). If everyone is going to reap the benefits of the new abundance brought on by advances in synthetic biology, then, policymakers must work on solving existing problems of access to medical care. To this end, scholars have hypothesized a number of ways in which the U.S. might improve access to care; offering health insurance to all its citizens, capping costs from co-payments and deductibles, and providing exemptions to out-of-pocket costs for high-value or high-need services are just a few examples (Sarnak et al., 2016). This approach should not only ensure that new medical technologies become widely accessible, but it should also do much to address a current significant source of inequality in the U.S.

In sum, what this analysis suggests is that ensuring equal access to newly abundant goods and services brought about by technology will require planning on the part of policymakers. The conventional rebuttal to any call for government intervention, of course, is that the invisible hand of the market will handle things most efficiently and so intervention should be stayed absent evidence of market failure. Here, I have tried to make the case that there is in fact market failure, rooted in the political and power dynamics underlying questions of access to and distribution of resources.

What, then, should this intervention look like? In some cases, it might involve the relatively straightforward step of ensuring that a particular technology like 3D printing is available in libraries or other facilities. In other cases, it will involve remedying existing structural inequalities and problems of access, including the current lack of access to medical services—a thorny and complex problem that demands a multi-pronged approach. But in any event, policymakers should not expect that the access issue will resolve itself, no matter what economics might predict, and they should be thinking now about how to implement policies that will help all citizens take advantage of newly abundant goods and services.

### Solving broader problems of inequality so that the new abundance can lead to real welfare gains

Planning to guarantee widespread access to a forthcoming abundance of goods and services is the first step in ensuring that this new abundance does not contribute to existing problems of inequality. But, even if successful, this planning will not mitigate or solve these problems. To be sure, ensuring widespread access to welfare-enhancing goods and services undoubtedly has the potential to improve lives. For example, a society in which more people have access to more advanced medical technologies is almost certainly better off than a society in which this access does not exist. But whether or not the new abundance is made available to all, in a society where other extreme forms of income and wealth inequality exist this abundance will not solve the myriad social problems that grow directly from inequality and its negative psychological impacts. In fact, depending on how policymakers respond, the new abundance could end up reinforcing the psychological distress that leads to this array of social problems seen in unequal societies. If policymakers wish to solve these problems, then, they need to tackle these other forms of inequality head-on, rather than expecting a new influx of widely available goods and services to do the work for them.

Exactly how they might do so is beyond the scope of this chapter, but many scholars have taken up the topic and offered a variety of innovative and feasible solutions. Further, lest the task seems too daunting, policymakers need not believe that achieving perfect equality—even if it were possible to do so—is necessary to reap the psychological and social benefits of more egalitarian societies. As discussed above, the psychological and social harms of inequality are often triggered by the sense of unfairness and mistrust that arise in situations of extreme and visible inequality that cannot be rationally justified. Indeed, there is at least some evidence that some level of justifiable inequality might be psychologically and socially beneficial because it gives people visible hope that they can improve their own situations in life (Cheung, 2016); but see Cheung (2016). Policymakers can therefore (at least initially) focus their efforts on addressing the extreme inequality that currently prevails in the U.S.; to this end, a number of proposed interventions, including inheritance and estate taxes, government transfers to bottom earners through universal basic income or earned income tax credits, and increased funding of social safety nets could be highly effective and lead to significant gains in the battle against inequality (Peterson Institute for International Economics, 2020).

## Conclusion

Society may soon experience a new abundance of goods and services as emerging technologies lower production and distribution costs. But the effect of this new abundance on current conditions of inequality in the U.S. has yet to be examined. Though it is tempting to hope that the coming abundance of goods and services will help remedy inequality and the social problems that attend it, my analysis here suggests that this prediction is unlikely to come to fruition without significant policy intervention. Instead, problems of inequality are likely to persist under new conditions of abundance, and in fact may worsen. For those interested in addressing the significant social problems that arise in unequal societies, the solution is two-fold. First, policymakers must plan for the coming abundance of goods and services in order to ensure that it is truly shared by all. And second, they must address extreme income, wealth, and other forms of inequality directly, rather than hoping, without basis, that increased access to goods and services will mitigate these social problems.

## Data availability statement

The original contributions presented in the study are included in the article/supplementary material, further inquiries can be directed to the corresponding author.

## Author contributions

The author confirms being the sole contributor of this work and has approved it for publication.

## Conflict of interest

The author declares that the research was conducted in the absence of any commercial or financial relationships that could be construed as a potential conflict of interest.

## Publisher's note

All claims expressed in this article are solely those of the authors and do not necessarily represent those of their affiliated organizations, or those of the publisher, the editors and the reviewers. Any product that may be evaluated in this article, or claim that may be made by its manufacturer, is not guaranteed or endorsed by the publisher.

## References

[B1] Alesina A. La Ferrara E. (2000). Participation in heterogeneous communities. Q. J. Econ. 115, 847–904. 10.1162/003355300554935

[B2] Algan Y. Cahuc P. (2013). Trust, Growth, and Well-Being: New Evidence and Policy Implications. (No. 7464). Bonn. Available online at: http://ftp.iza.org/dp7464.pdf (accessed July 15, 2022).

[B3] Allcott H. Diamond R. Dubé J.-P. Handbury J. Rahkovsky I. Schnell M. (2018). Food Deserts and the Causes of Nutritional Inequality. NBER Working Paper Series, 1–78.

[B4] Bakkeli N. Z. (2020). Health and economic scarcity: measuring scarcity through consumption, income and home ownership indicators in Norway. SSM Popul. Health 11, 1–16. 10.1016/j.ssmph.2020.100582 32322658 PMC7171528

[B5] Bell L. A. Freeman R. B. (2001). The incentive for working hard: explaining hours worked differences in the US and Germany. Labour Econ. 116, 447–487. 10.3386/w8051

[B6] Bowles S. Park Y. (2005). Emulation, inequality, and work hours. Was Thorsten Veblen Right? Econ. J. 115, 397–415. 10.1111/j.1468-0297.2005.01042.x

[B7] Buttrick N. R. Oishi S. (2017). The psychological consequences of income inequality. Soc. Pers. Psychol. Compass 11, 1–12. 10.1111/spc3.12304

[B8] Chattergee E. (2019). A climate of scarcity: electricity in India, 1899-2016, in Scarcity in the Modern World: History, Politics, Society and Sustainability 1800-2075, eds Jonsson F. A. Brewer J. Fromer N. Trentmann F. (London: Bloomsbury), 211–228.

[B9] Cheung F. (2016). Can income inequality be associated with positive outcomes? Hope mediates the positive inequality-happiness link in Rural China. Soc. Psychol. Pers. Sci. 7, 320–330. 10.1177/1948550615619762

[B10] Cheung F. Lucas R. E. (2016). Income inequality is associated with stronger social comparison effects: the effect of relative income on life satisfaction. J. Pers. Soc. Psychol. 110, 332–341. 10.1037/pspp0000059 26191957 PMC4718872

[B11] Coccia M. (2018). Economic inequality can generate unhappiness that leads to violent crime in society. Int. J. Happ. Dev. 4, 1–24. 10.1504/IJHD.2018.10011589

[B12] Costa D. Kahn M. (2003). Understanding the decline in American social capital, 1953-1998. Kyklos 56, 17–46. 10.1111/1467-6435.00208

[B13] Crawford S. (2018). Fiber: The Coming Tech Revolution—And Why America Might Miss It. New Haven, CT: Yale.

[B14] DeCelles K. A. Norton M. I. (2016). Physical and situational inequality on airplanes predicts air rage. PNAS 113, 5588–5591. 10.1073/pnas.1521727113 27140642 PMC4878482

[B15] Desai D. R. Magliocca G. N. (2014). Patents, meet napster: 3D printing and the digitization of things. Georgetown Law Rev. 102, 1691–1720.

[B16] Elgar F. J. (2010). Income inequality, trust, and population health in 33 countries. Am. J. Public Health 100, 2311–2315. 10.2105/AJPH.2009.189134 20864707 PMC2951926

[B17] Elgar F. J. Aitken N. (2011). Income inequality, trust and homicide in 33 countries. Eur. J. Public Health 21, 241–346. 10.1093/eurpub/ckq068 20525751

[B18] Fairfield J. (2022). Property as the Law of Virtual Things. 10.3389/frma.2022.981964 PMC9458901 36091970

[B19] Ferrer-i-Carbonell A. Ramos X. (2012). Inequality and Happiness: A Survey. GINI Discussion Paper 2012. Available online at: http://www.gini-research.org/system/uploads/374/original/DP_38_-_Ferrer-i-Carbonell_Ramos.pdf (accessed September 9, 2021).

[B20] Frank R. (2015). Most Millionaires Say They're Middle Class. New York, NY: CNBC.

[B21] Gino F. Pierce L. (2009). The abundance effect: unethical behavior in the presence of wealth. Org. Behav. Hum. Decision Proc. 109, 142–155. 10.1016/j.obhdp.2009.03.003

[B22] Grosch K. Rau H. A. (2020). Procedural unfair wage differentials and their effects on unethical behavior. Econ. Inquiry 58, 1689–1706. 10.1111/ecin.12906

[B23] Grosfeld I. Senik C. (2010). The emerging aversion to inequality. Econ. Trans. 18, 1–26. 10.1111/j.1468-0351.2009.00376.x

[B24] Jonsson F. A. Brewer J. Fromer N. Trentmann F. (eds.) (2019). Scarcity in the Modern World: History, Politics, Society and Sustainability 1800-2075. London: Bloomsbury.

[B25] Koh S. Y. (2020). Inequality, in International Encyclopedia of Human Geography, eds Castree N. Crang M. Domosh M. (Kingston, ON: Elsevier).

[B26] Kolbert E. (2018). The Psychology of Inequality. New York, NY: New Yorker.

[B27] Kraus M. W. Tan J. J. X. Tannenbaum M. B. (2013). The social ladder: a rank-based perspective on social class. Psychol. Inquiry 24, 81–96. 10.1080/1047840X.2013.778803

[B28] Lamoureux D. (2019). Lagos ‘scarce-city’: investigating the roots of urban modernity in a colonial capital, 1900-1928, in Scarcity in the Modern World: History, Politics, Society and Sustainability 1800-2075, eds Jonsson F.A. Brewer J. Fromer N. Trentmann F. (London: Bloomsbury), 229–246.

[B29] Lancee B. van der Werfhorst H. (2011). Income Inequality and Participation: A Comparison of 24 European Countries. (No. 6). Amsterdam: Social Science Research, Elsevier. 10.1016/j.ssresearch.2012.04.005 23017925

[B30] Layte R. Whelan C. T. (2014). Who feels inferior? A test of the statis anxiety hypothesis of social inequalities in health. Eur. Sociol. Rev. 30, 525–535. 10.1093/esr/jcu057

[B31] Lemley M. A. (2015). IP in a world without scarcity. New York Univ. Law Rev. 90, 460–515. 10.31235/osf.io/3vy5a

[B32] Merriam-Webster (2020). Scarce. In Merriam-Webster.com dictionary. Available online at: https://www.merriam-webster.com/dictionary/scarce (accessed September 2, 2021).

[B33] Messias E. Eaton W. W. Grooms A. N. (2011). Income inequality and depression prevalence across the United States: an ecological study. Psychiatric Serv. 62, 710–712. 10.1176/ps.62.7.pss6207_0710 21724781

[B34] Millman M. (ed.). (1993). Access to Health Care in America. Washington, DC: National Academy. 25144064

[B35] Mirsha S. Hing L. S. S. Lalumière (2015). Inequality and risk-taking. Evol. Psychol. 13, 1–11. 10.1177/1474704915596295 37924188 PMC10426876

[B36] Neville L. (2012). Do economic inequality and generalized trust inhibit academic dishonesty? Evidence from state-level search-engine queries. Psychol. Sci. 23, 339–345. 10.1177/0956797611435980 22421204

[B37] Nishi A. Shirado H. Rand D. G. Christakis N. A. (2015). Inequality and visibility of wealth in experimental social networks. Nature 526, 426–429. 10.1038/nature15392 26352469

[B38] Organ D. W. Moorman R. H. (1993). Fairness and organizational citizenship behavior: what are the connections? Soc. Justice Res. 6, 5–18. 10.1007/BF01048730

[B39] Osborn R. Squires D. Doty M. M. Sarnak D. O. Schneider E. C. (2016). In a New Survey of 11 Countries, U.S. Adults Still Struggle with Access to and Affordability of Health Care. Health AffairsWeb First. Available online at: https://www.commonwealthfund.org/sites/default/files/documents/___media_files_publications_in_the_literature_2016_nov_1915_osborn_2016_intl_survey_ha_11_16_2016_itl_v2.pdf (accessed July 15, 2022). 10.1377/hlthaff.2016.1088 27856648

[B40] Palazzolo M. Pattabhiramaiah A. (2020). The Minimum wage and consumer nutrition (J. Marketing Research), 1–78.

[B41] Paskov M. Gërxhani K. van de Werfhorst H. G. (2013). Income Inequality and Status Anxiety. “Amsterdam” AIAS, GINI Discussion Paper 90.s

[B42] Payne K. (2017). The Broken Ladder: How Inequality Affects the Way We Think, Live, And Die. New York, NY: Viking.

[B43] Peterson Institute for International Economics (2020). How to Fix Economic Inequality? An Overview of Policies for the United States and Other High-Income Economies. Available online at: http//www.piie.com/sites/default/files/documents/how-to-fix-economic-inequality.pdf (accessed July 15, 2022).

[B44] Piketty T. (2014). Capital in the Twenty-First Century. Cambridge, MA: Belknap. 10.1111/1468-4446.12115 25516350

[B45] Sarnak D. A. Doty M. M. Squires D. Osborn R. (2016). Fewer Americans Say Cost is a barrier to Getting Care, But U.S. Still Has a Long Way to Go. Health Affairs Web First. Available online at: https://www.commonwealthfund.org/blog/2016/fewer-americans-say-cost-barrier-getting-care-us-still-has-long-way-go (accessed July 15, 2022).

[B46] Sen A. (1981). Poverty and Fairness: An Essay on Entitlement and Deprivation. Oxford: Clarendon.

[B47] Uslaner E. M. Brown M. (2005). Inequality, trust, and civic engagement. Am. Politics Res. 33, 868–894. 10.1177/1532673X04271903

[B48] Walasek L. Brown G. D. A. (2015). Income inequality and status seeking: searching for positional goods in unequal U.S. States. Psychol. Sci. 26, 527–533. 10.1177/0956797614567511 25792131

[B49] Walker R. E. Keane C. R. Burke J. G. (2010). Disparities and access to healthy food in the united states: a review of food desert literature. Health Place 16, 876–884. 10.1016/j.healthplace.2010.04.013 20462784

[B50] Wilkinson R. Pickett K. (2018). The Inner Level: How More Equal Societies Reduce Stress, Restore Sanity, and Improve Everyone's Well-Being. New York, NY: Penguin. 10.3399/bjgp19X705377 PMC6715479 31467020

[B51] Wilkinson R. G. Pickett K. E. (2009). Income inequality and social dysfunction. Ann. Rev. Sociol. 35, 493–511. 10.1146/annurev-soc-070308-115926

[B52] Yang F. Shaw A. Garduno E. Olson K. R. (2014). No one likes a copycat: a cross-cultural investigation of children's response to Plagiarism. J. Exp. Psychol. 121, 111–119. 10.1016/j.jecp.2013.11.008 24473471

